# Ketogenic Diet-Induced Severe Ketoacidosis in a Lactating Woman: A Case Report and Review of the Literature

**DOI:** 10.1155/2019/1214208

**Published:** 2019-07-08

**Authors:** Benedicta Nneoma Nnodum, Eziafa Oduah, David Albert, Mark Pettus

**Affiliations:** Berkshire Medical Center, Pittsfield, MA 01201, USA

## Abstract

The ketogenic diet (KD) is a high-fat, adequate-protein, and low-carbohydrate diet that leads to nutritional ketosis and weight loss. It is known to induce ketosis but is not an established cause of clinically significant ketoacidosis. Lactation ketoacidosis is well established in bovine literature but remains a rare phenomenon in humans. Here we present a life-threatening case of severe ketoacidosis in a nondiabetic lactating mother on a strict ketogenic diet. We review the available case reports of lactation ketoacidosis in humans and the mechanisms thereof. Although ketogenic diet has been shown to be safe in nonpregnant individuals, the safety of this diet in lactating mothers is not known. Health professionals and mothers should be made aware of the potential risk associated with a strict ketogenic diet when combined with lactation. Prompt diagnosis and immediate treatment cannot be overemphasized. To our knowledge, this is the first reported case of life-threatening lactation ketoacidosis associated with ketogenic diet while consuming an adequate number of calories per day.

## 1. Introduction

Ketogenic diet is a high-fat, adequate-protein, and low-carbohydrate diet that leads to nutritional ketosis and weight loss. Although ketogenic diet is safe in nonpregnant individuals, its safety in lactating mothers is unknown. This case report aims to increase the awareness to healthcare professionals, mothers, and dietitians on the potential risk associated with a strict ketogenic diet when combined with lactation.

## 2. Case Presentation

A healthy nondiabetic 24-year-old 18 weeks postpartum woman presented to the emergency department with severe nausea, vomiting, and several episodes of diarrhea of 9-hour duration. While in the emergency department, she developed abdominal pain, low back cramps, and malaise. The patient reported adhering to a strict ketogenic diet as a health-conscious life style modification since the recent birth of her 18-week-old son. She had continued to provide her son an exclusively breastfed diet since birth. She reported an intentional 25-pound weight loss in the 18-week postpartum period. She had an unremarkable prenatal care and delivered a healthy baby boy at 40 weeks of gestation by spontaneous vaginal delivery. Her puerperium was otherwise uneventful. She denied smoking and use of alcohol. Dietary review revealed a typical breakfast consisting of egg and bacon; lunch usually consisted of some variation of vegetable salad with cheese, and a dinner consisted of vegetables and meat. Typical food items include vegetables such as peppers, spinach, broccoli, cheese, and carrot soups. For protein, she consumed mostly chicken, salmon, and other white fish. She avoided nuts or shellfish but would have 1-2 tablespoons of peanut butter per day. She eliminated all other forms of carbohydrate including wheat, pasta, bread, or any grain products. She denied any restriction in her daily caloric intake, stating that she consistently tracked her macronutrients at an average of 2200 Kcals per day.

On presentation to the emergency department, her blood pressure was 117/82 mmHg, heart rate of 103 beats/min, respiratory rate of 18 cycles/min, a temperature of 98.1°C, and oxygen saturation 98% on room air. Physical examination was remarkable for dry mucous membranes, comfortable resting tachypnea though she was breathing more deeply, mild epigastric/right upper quadrant tenderness, but was otherwise unremarkable.

**Figure 1:**
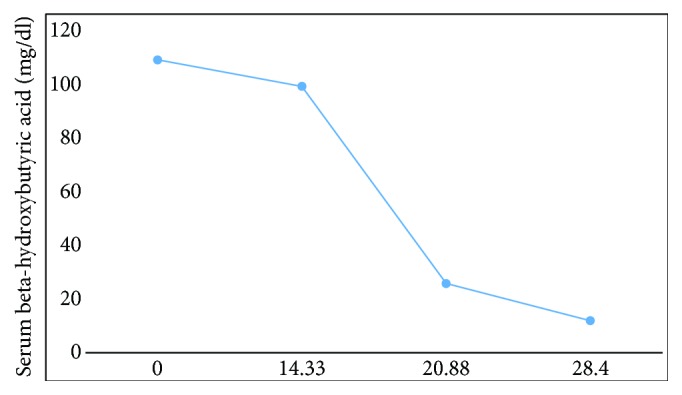
Trend of serum beta-hydroxybutyric acid level during hospitalization.

Laboratory studies revealed a chemistry panel with sodium of 138 meq/L; potassium, 4.3 meq/L; chloride, 109 meq/L; urea, 10 mg/dl; creatinine, 0.84 mg/dl; bicarbonate, 6 meq/L; glucose 68mg/dl; calculated anion gap of 27.3 meq/L; phosphorus of 2.3meq/L; calcium of 9.7mg/dl; and venous lactate of 1.3 mmol/L. Hematologic indices were grossly unremarkable with hemoglobin of 14.4 g/dl; white blood cells at 9.4; and neutrophils, 7.8. Her beta-hydroxybutyric acid level was initially markedly elevated at 109.5mg/dl (Figure 1). Urinalysis revealed trace protein, ketones 4+, and hemoglobin A1c, 4.8%. Osmolar gap was normal. An arterial blood gas evaluation showed a compensated AG metabolic acidosis acidaemia with pH- 7.11; partial pressure of carbon dioxide, 17 mmHg; bicarbonate (HCO_3_), 5.3 mmol/L; base excess, -22.2 mmol/L. Electrocardiogram revealed normal sinus rhythm with a rate of 87 and prolonged QTc interval of 506. Ultrasound of the abdomen was unremarkable. Toxicology screen was negative except for cannabinoids.

She was initially managed with a combination of IV fluids including NS, Isotonic Bicarbonate, and D5W. A repeat arterial blood gas in six hours showed improved pH- 7.28; partial pressure of carbon dioxide, 15.8 mmHg; bicarbonate (HCO_3_), 7.8 mmol/L; base excess, -16.9 mmol/L. She was immediately restarted on oral carbohydrate diet. She was allowed to continue to breastfeed her infant as she preferred. She received insulin with glucose supplementation as part of the treatment of nondiabetic ketoacidosis. Insulin levels measured at different times during hospitalization showed appropriate response to blood glucose levels ruling out euglycemic diabetic ketoacidosis. During her treatment, she developed significant metabolic derangements including worsening hypophosphatemia down to 1.0, hypokalemia to a nadir of 2.6, and hypocalcemia down to 7.3. These were closely monitored and were repleted accordingly. She experienced associated symptoms of digital tingling, perioral anesthesia, and trousseau syndrome which gradually improved with therapy. Interestingly, she did not develop respiratory failure associated with severe hypophosphatemia.

With the initiation of carbohydrates and dextrose her blood B-hydroxybutyric acid normalized and her anion gap closed in less than 24 hours of hospitalization. She was successfully discharged on day 4 of hospitalization (Table 2) with close nephrology and primary care follow-up and symptom resolution. Follow-up labs after discharge remained normal (Table 2).

## 3. Discussion

There are several established causes of ketoacidosis including diabetic ketoacidosis, starvation ketoacidosis, alcoholic ketoacidosis, and some inborn errors of metabolism including succinyl CoA:3 ketoacid CoA transferase deficiency, mitochondrial 2-methylacetoacetyl CoA thiolase deficiency, and methylmalonyl-CoA mutase deficiency [1]. Lactation ketoacidosis is well described in postpartum lactating cattle in the veterinary literature [2]. However, only a few case reports of lactation ketoacidosis in human have been reported to date. Most of these cases were precipitated by situations of starvation or infection requiring lactating mothers to be nil per mouth (Table 1). To our knowledge, this is the first case report of this uncommon phenomenon in humans in the setting of a ketogenic diet with adequate number of calories, above 2000 kcal/day.

Ketogenic diet (KD) is described as a high-fat, adequate-protein, and low-carbohydrate diet (<50g/day) [3]. It has been used as an alternative weight loss tool against obesity due to proven results of greater weight loss compared to other balanced diets [4, 5]. The underlying mechanism appears to culminate in nutritional ketosis induced by the low availability of carbohydrate as the body is forced to burn fats instead of carbohydrate for energy metabolism. This process involves the conversion of fats by the liver into fatty acids thus producing ketone bodies. The resultant effect is the accumulation of ketones in blood and urine but to a level not associated with changes in blood pH, which is the hallmark of life-threatening ketoacidosis as seen in such conditions as diabetic ketoacidosis [3]. The importance of the ketogenic diet can be traced back to the 1920s [6]. It is one of the most studied interventions for weight loss as it has a physiological and biochemical basis to induce effective weight loss [7].

We came across few studies that evaluated the acid-base safety of patients on ketogenic diet, one showed that majority had a ketone level less than 54mg/dl and none was greater than 106 mg/dl while the other study reported patients had a mild compensated metabolic acidosis with no significant metabolic derangement [8, 9]. Studies carried out by Mohammed et al. showed that healthy lactating women on low-carbohydrate diet or 42-hour fast had a significantly increased hydroxybutyric acid compared with nonlactating women due to decreased carbohydrate oxidation and increased fat oxidation [10, 11]. This condition commonly referred to as “bovine ketosis” is common in dairy cows when they are unable to compensate for high energy requirements of milk production by sufficient energy intake [12].

Postpartum mothers are at an increased pressure to lose weight gained during pregnancy and may resort to this proven efficacious method of rapid weight loss. The increased energy demands of lactation which results in increased glucose utilization may lead to a dysregulation of the compensatory mechanism regulating normal ketone levels in lactating women on ketogenic diet [13]. Lactation ketoacidosis can occur by adhering to a low-carbohydrate diet which leads to depletion of the glycogen stores forcing the body into using gluconeogenesis as an energy substrate for production of breast milk [1].

Existing case reports like ours demonstrated complete resolution of symptoms, ketoacidosis, and laboratory derangements with rehydration and restarting normal diet.

## 4. Conclusion

Although ketogenic diet is being considered a safe nutritional intervention for weight loss and has grown to be a popular implementation for successful weight loss in the medical literature, social media, and fitness blogs, the index case may provide caution in lactating mothers on/or considering a ketogenic diet. It is important for clinicians to educate lactating mothers interested in weight.

## Figures and Tables

**Table 1:** Literature review of existing case reports and their precipitating factor.

Case Report	Precipitating Factor
A case of lactation “bovine” ketoacidosis [14]	Breastfeeding twins in the setting of a “selected diet”

A severe case of iatrogenic lactation ketoacidosis [15]	Nil per oral for 3 days to treat a bowel obstruction

“Bovine ketosis” in a nondiabetic postpartum woman [16]	Urinary tract infection in the setting of a weight reduction diet

Severe spontaneous “bovine” ketoacidosis in a lactating woman [17]	Urinary tract infection in the setting of a high protein, carbohydrate-free reduction diet

A case of bovine ketoacidosis in a lactating woman [18]	2-day nausea & vomiting in the setting of several small high-protein carbohydrate-free meals

Ketoacidosis associated with low-carbohydrate diet in a non-diabetic lactating woman [19]	Low carbohydrate, high fat diet for 10 days

Starvation ketosis in a breastfeeding woman [20]	Bariatric surgery during lactation

Severe ketoacidosis in breastfeeding woman with low energy and carbohydrate intake [21]	Illness while on a low carbohydrate diet

Ketoacidosis in a non-diabetic woman who was fasting during lactation [22]	Starvation during lactation due to abdominal pain

Lactation ketoacidosis: an unusual entity and a review of the literature [23]	No precipitating factor except for lactation

Life-threatening lactation or Bovine ketoacidosis [24]	Frequent skipping of meals while on a high protein, low carbohydrate diet

A rare cause of metabolic acidosis: ketoacidosis in a non-diabetic lactating woman [25]	Gastroenteritis in the setting of a low carbohydrate diet

**Table 2:** Table demonstrating serial pertinent laboratory values during hospitalization and after discharge.

	On admission, June 15^th^	June 16^th^	June 17^th^	June 18^th^	On discharge, June 19^th^	1 week after discharge, June 26^th^
Serum bicarbonate (meq/L)	6	10	24	30	29	23

Glucose (mg/dl)	50	124	112	105	99	77

Insulin level (uiu/ml)	3.4	11.1				

Phosphorous (mg/dl)	2.3	1.4	1.0	2.4	3.3	3.9

Potassium (meq/l)	4.2	3.0	2.6	3.1	4.3	4.0

Calcium (mg/dl)	7.3	7.9	8.8	8.6	9.2	8.8

Urine Ketone	4+					

pH ABG	7.11	7.28				

pCO_2_ ABG	17.0	15.8				

Bicarbonate ABG	5.3	7.3				

ABG: arterial blood gas.
